# Preparation, Characterization, and Electrochemical Performance of the Hematite/Oxidized Multi-Walled Carbon Nanotubes Nanocomposite

**DOI:** 10.3390/molecules27092708

**Published:** 2022-04-22

**Authors:** Hadeel M. Banbela, Laila M. Alharbi, Reema H. Al-Dahiri, Mariusz Jaremko, Mohamed Abdel Salam

**Affiliations:** 1 Department of Chemistry, Faculty of Science, King Abdulaziz University, P.O. Box 80200, Jeddah 21589, Saudi Arabia; hbanbela@stu.kau.edu.sa (H.M.B.); lalhrbi@kau.edu.sa (L.M.A.); 2 Department of Chemistry, College of Science and Arts at Khulis, University of Jeddah, P.O. Box 355, Jeddah 21959, Saudi Arabia; 3 Department of Chemistry, College of Science, University of Jeddah, P.O. Box 34, Jeddah 21959, Saudi Arabia; rhal-dhahery@uj.edu.sa; 4 Smart-Health Initiative (SHI), Red Sea Research Center (RSRC), Biological and Environmental Science and Engineering (BESE) Division, King Abdullah University of Science and Technology (KAUST), P.O. Box 4700, Thuwal 23955-6900, Saudi Arabia; mariusz.jaremko@kaust.edu.sa

**Keywords:** hematite, nanoparticle, MWCNT, electrochemical performance, capacitive behaviour

## Abstract

In this research work, a hematite (α-Fe_2_O_3_) nanoparticle was prepared and then mixed with oxidized multi-walled carbon nanotubes (O-MWCNT) to form a stable suspension of an α-Fe_2_O_3_/O-MWCNTs nanocomposite. Different characterization techniques were used to explore the chemical and physical properties of the α-Fe_2_O_3_/O-MWCNTs nanocomposite, including XRD, FT-IR, UV-Vis, and SEM. The results revealed the successful formation of the α-Fe_2_O_3_ nanoparticles, and the oxidation of the MWCNT, as well as the formation of stable α-Fe_2_O_3_/O-MWCNTs nanocomposite. The electrochemical behaviour of the α-Fe_2_O_3_/O-MWCNTs nanocomposite was investigated using cyclic voltammetry (CV) and linear sweep voltammetry (LSV), and the results revealed that modification of α-Fe_2_O_3_ nanoparticles with O-MWCNTs greatly enhanced electrochemical performance and capacitive behaviour, as well as cycling stability.

## 1. Introduction

Nanotechnology is a general term focused on the manipulation and applications of nanoparticles (NPs), such as metals, metal oxides, semiconductors, ceramics, and polymers, due to their outstanding structural, physicochemical and morphological properties, which allows them to be used in a wide variety of applications, especially in the energy sector. For example, many metal oxides are used as electrodes for lithium-ion batteries (LIBs) due to their outstanding and efficient surface area, as well as for their chemical suitability and stability, as they intercalate/deintercalate lithium ions into their layered structure [1,2,3], which significantly enhances LIB storage capacities even beyond their theoretical values [4]. Hematite (α-Fe_2_O_3_) nanostructures are one of the promising metal oxide nanoparticles which have attracted the attention of researchers and are used for a variety of applications, including splitting of water and the production of hydrogen gas [5], magnetic storage devices [6], potential gas sensors [7], targeted drug delivery [8], and biomedical applications [9], as well as LIB production [10,11,12,13].

Oxygen evolution reaction (OER) has become the common power source for sustainable energy development technology, especially the photoelectrochemical (PEC) cells, which use solid-state electrodes in a similar way to conventional electrolysers such as in oxygen evolution reactions (OER, oxidation) and hydrogen evolution reactions (HER, reduction), which take place at two different solid/liquid junctions. In PEC cells, at least one of the electrodes consists of a semiconductor capable of absorbing the incoming light, in which a depletion (or space-charge, SC) region is formed at equilibrium, and the photogenerated charges are separated by the electric field in the SC region and travel to a solid/liquid junction where they take part in either the HER or the OER. The electro-catalysts of non-noble metals have been interesting in this field; reportedly, Hematite has an outstanding performance of oxygen evolution reaction (OER), with an optical narrow bandgap around ~2.2 eV, which absorbs light up to 560 nm and which allows it to absorb 40% of solar irradiance. Hematite is a naturally abundant, low-cost material, and has good chemical stability in aqueous solutions in a broad pH range. The position of the valence band is suitable for oxygen evolution, which makes it an ideal candidate photoanode material for solar water splitting [14,15]. Conversely, hematite has shown low efficiency because of its poor electrical conductivity. The critical reasons are high recombination of electrons and holes, low mobility of the holes/short holes diffusion length and trapping of electrons by oxygen deficiency sites. Various techniques are reported in the literature to improve the efficiency of hematite by inserting the effective materials in the lattice without disturbing the structure of the crystal. Enhancing the efficiency of hematite for photoelectrochemical water splitting was carried out by doping with elements/ions such as Ti^4+^ [16], manganese [17], zirconium and tin [18], tantalum [19], boron [20], phosphorous [21], rhodium [22], and tetravalent dopants (Si^4+^, Sn^4+^, Ti^4+^, and Zr^4+^) [23]. Few works have been dedicated to doping with carbon-based materials such as carbon dots [24,25] and graphene [26,27]. Although, one of the computational studies showed that the water splitting of hematite could be significantly improved by forming composites with carbon nanotubes [28]; the number of studies focusing on this topic is scarce in the literature [29].

In this work, the electrochemical behaviour of the hematite/oxidized multi-walled carbon nanotubes (O-MWCNT) nanocomposite was studied and explored. First, hematite was prepared using the hydrothermal method and was then mixed with the oxidized MWCNTs to form a stable nanocomposite. The hematite/O-MWCNTs nanocomposite was then characterized by XRD in order to explore the chemical and physical characteristics. The electrochemical behaviour of the O-MWCNT/hematite nanocomposite were investigated using cyclic voltammetry (CV) and linear sweep voltammetry (LSV) to explore their possible applications.

## 2. Experimental

### 2.1. Chemicals and Materials

Ferric chloride hexahydrate (FeCl_3_·6H_2_O) was obtained from Lobachemie and was used as received without further purification. Multi-walled carbon nanotubes (MWCNT) were provided from Sigma-Aldrich (St. Louis, MI, USA). Sulfuric acid—95–99% (H_2_SO_4_) and nitric acid—65% (HNO_3_) were purchased from Chem-Lab. (Bunkyo, Tokyo). Potassium hydroxide (KOH) was obtained from Fluka Chemie AG (Buchs, Switzerland). All aqueous solutions were prepared with distilled water.

### 2.2. Preparation of Hematite (α-Fe_2_O_3_) Nanoparticles

The hematite nanoparticles were prepared according to the procedure used by Faust et al. [30]. Initially, 30 mL solution of 0.1 M FeCl_3_·6H_2_O was dissolved in distilled water and added dropwise into 120 mL stirred boiling water; subsequently, the solution was refluxed for 5 min and finally cooled in an ice bath. The colloidal α-Fe_2_O_3_ suspension had a dark red colour and was acidic:

2 FeCl_3_ + 3 H_2_O → α-Fe_2_O_3_ + 6 HCl

The colloid was dialyzed by using a dialysis tube (Medicell International, MWCO 12,000–14,000) in the distilled water, which changed several times until the pH had reached pH~6 and the electrical conductivity was below 20 S cm^−1^. The product was kept in the dark.

### 2.3. Oxidation of MWCNT

The purification and oxidation of MWCNTs were performed as follows: 1.0 g of the pristine MWCNTs was added to 200 mL solution of concentrated HNO_3_/H_2_SO_4_ (1:3; *v*/*v*) in an ice bath. The mixture was sonicated for 4 h in an ultrasonic bath at 40 °C. The resulting solution was then transferred to a 1000 mL beaker with distilled water to reduce the acidity of the product, which was then neutralized by using a dialysis tube (Medicell International, MWCO 12,000–14,000) in the distilled water, that were changed several times until achieving a neutral pH.

### 2.4. Preparation of α-Fe_2_O_3_/O-MWCNTs Nanocomposite

The hematite was deposited onto the surface of MWCNTs by mixing at RT for one hour under ultrasonication with different concentrations of MWCNT.

All prepared suspensions before and after mixing were dried overnight in an oven at 50 °C. For further characterisation, the solutions and the powder were obtained in the dark.

For XRD measurements, the resulting powder was calcined at 200 °C for 2 h at a heating rate of 5 °C min^−1^ in a preheated muffle furnace.

### 2.5. Electrode Fabrication

The α-Fe_2_O_3_ and α-Fe_2_O_3_/O-MWCNTs electrodes were prepared for electrochemical investigations. Approximately 50 μL of the prepared suspensions was dropcasted upon the glassy carbon electrode (GCE) in a rotating ring disk electrode system (RRDE), which was used as a working electrode.

### 2.6. Characterization

The optical absorptions of the prepared hematite with different pH values were investigated by using a MultiSpec-1501 UV–Vis Spectrophotometer (SHIMADZU). The determination of the functional group on the surface of the MWCNTs was performed with a PerkinElmer Spectrum 100 infrared spectrometer (FTIR spectra), where the dried samples were mixed with potassium bromide (ratio of 1:10) and pressurized to produce KBr pellet for FTIR measurements. The identification of crystalline phases of the samples was recorded using a Bruker D2 Phaser X-ray diffractometer. The XRD measurements were carried out by CuKα radiation (1.5418 Å). The XPS experiments were performed on a Kratos Axis Ultra DLD instrument equipped with a monochromatic Al Kα X-ray source (hν = 1486.6 eV) operating at a power of 75 W and under UHV conditions in the range of ∼10^−9^ mbar. All spectra were recorded in hybrid mode using electrostatic and magnetic lenses and an aperture slot of 300 × 700 μm. The survey and high-resolution spectra were acquired at fixed analyser pass energies of 160 and 20 eV, respectively. The samples were mounted in floating mode in order to avoid differential charging. Thereafter, XPS spectra were acquired using charge neutralization. The XPS experiments were performed on a Kratos Axis Ultra DLD instrument equipped with a monochromatic Al Kα X-ray source (hν = 1486.6 eV) operating at a power of 75 W and under UHV conditions in the range of ∼10^−9^ mbar. All spectra were recorded in hybrid mode using electrostatic and magnetic lenses and an aperture slot of 300 × 700 μm. The survey and high-resolution spectra were acquired at fixed analyser pass energies of 160 and 20 eV, respectively. The samples were mounted in floating mode in order to avoid differential charging. Thereafter, XPS spectra were acquired using charge neutralization.

### 2.7. Electrochemical Measurements

The electrochemical measurements included cyclic voltammetry (CV) and linear sweep voltammetry (LSV), and the electrochemical impedance spectroscopy (EIS) studies were performed using the CorrTest electrochemical workstation with an RRDE three-electrode system. The working electrodes were fabricated by dropcasting a sample as described in the experimental Section 2.5. The Ag/AgCl was the reference electrode and Pt wire the counter electrode in 1.0 M KOH as the electrolyte. CV and LSV curves of α-Fe_2_O_3_ were recorded at a potential of 100 Hz and with a scan rate of 100 mV s^–1^.

## 3. Results and Discussion

### 3.1. Characterization

**Figure 1:**
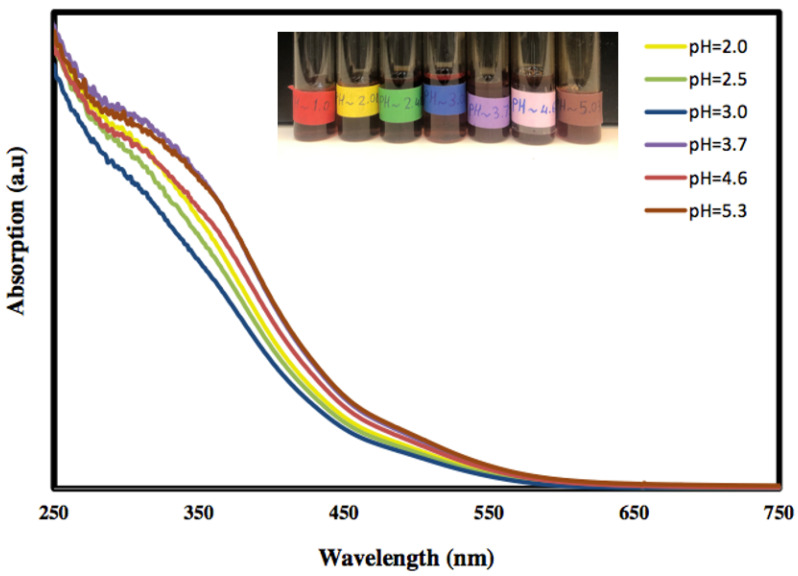
The UV–Visible absorption curves of hematite nanoparticles at different pH values.

The optical properties of the freshly prepared α-Fe_2_O_3_ nanoparticles at various pH values were investigated using UV–Vis absorption spectroscopy, and the results are presented in Figure 1. It is clear that the absorption of the colloidal α-Fe_2_O_3_ begins below 560 nm, indicating that the prepared α-Fe_2_O_3_ is a visible-light-active photocatalyst. Moreover, the absorption below 560 nm is due to the absorption of shorter wavelengths of the visible region (yellow to ultraviolet photons), and the good transmission of red light which yields the characteristic red colour of hematite.

**Figure 2:**
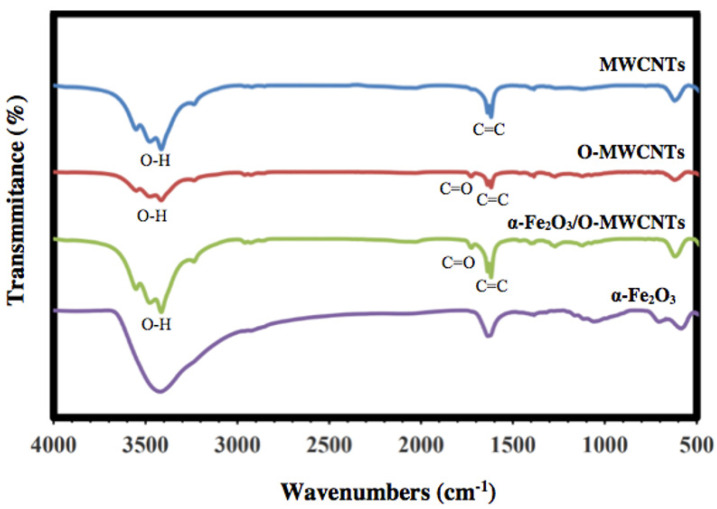
FT-IR spectra of MWCNTs, oxidized MWCNTs, hematite nanoparticles, and hematite/O-MWCNTs nanocomposite.

FT-IR was used to investigate the successful oxidation of the MWCNTs and preparation of α-Fe_2_O_3_, as well as the α-Fe_2_O_3_/O-MWCNTs nanocomposite, and the results are shown in Figure 2. The FTIR spectrum of the pristine MWCNTs showed the characteristic vibration peaks at 1680–1640 cm^−1^ (C=C stretch) and 1500–1400 cm^−1^ (C-C stretch) due to the carbon hexagonal ring of the MWCNT. In contrast, the oxidized MWCNTs (O-MWCNT) spectrum showed a clear and strong absorption peak at 1730 cm^−1^ and a weak peak at 1130 cm^−1^, which could be attributed to the stretching vibration of C=O corresponding to the stretching vibration of C=O from the carboxylic acid groups (-COOH) and C-O stretching from either a phenol or lactone, which confirmed the successful oxidation of the MWCNTs [31]. The FTIR spectrum of α-Fe_2_O_3_ showed characteristic Fe-O sharp peaks at 478 and 568 cm^−1^ due to the vibrational mode [32]. Furthermore, the FTIR spectrum of the α-Fe_2_O_3_/O-MWCNTs nanocomposite showed three new peaks in the range of 1760–1100 cm^−1^, which appeared after the mixing of α-Fe_2_O_3_ with O-MWCNT, and band shifting occurred from 600 to 628 cm^−1^, which indicated that the hematite surface was modified by the oxidized MWCNT. A broad peak at approximately 3500 cm^−1^ for all the investigated samples could be assigned to O-H stretching, due to moisture from the environment, and/or alcoholic, phenolic, or carboxylic groups at the oxidized MWCNTs surface due to oxidation.

**Figure 3:**
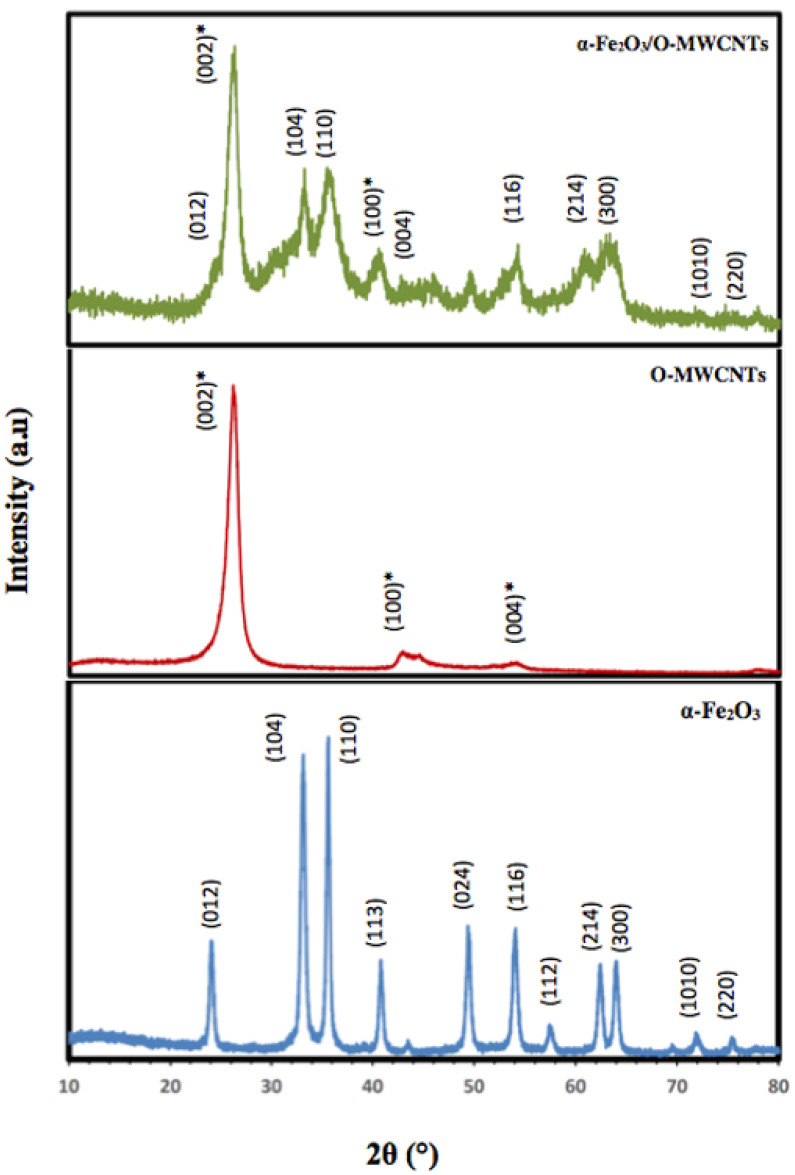
XRD of hematite nanoparticles, oxidized MWCNTs, and hematite/O-MWCNTs nanocomposite (* for the O-MWCNTs).

Figure 3 illustrates the XRD patterns of the prepared α-Fe_2_O_3_ nanoparticles, O-MWCNTs and the α-Fe_2_O_3_/O-MWCNTs nanocomposite. According to the XRD, the characteristic peaks of the hexagonal crystal system α-Fe_2_O_3_ nanoparticles were identified from the diffraction peaks at 2θ = 24.20°, 33.21°, 35.70°, 40.92°, 49.60°, 54.15°, 57.90°, 62.50°, and 64.10°, related to (012), (104), (110), (113), (024), (116), (112), (214), and (300), respectively (JCPDS card no 96-900-9783). Moreover, the sharp peaks of the α-Fe_2_O_3_ nanoparticles indicate the highly crystalline structure of the hexagonal crystal system, with an average crystallite size of 18.55 nm, as calculated from the Scherer equation. Conversely, O-MWCNTs exhibits two diffraction peaks: one at 26.14° (002 plane) and the other at 44.22° (100 plane), corresponding to graphitic carbon (JCPDS card no 89-8487). Furthermore, the XRD pattern of the α-Fe_2_O_3_/O-MWCNTs nanocomposite exhibits the characteristic peaks of both the hexagonal crystal system of α-Fe_2_O_3_ nanoparticles and the oxidized MWCNTs.

**Figure 4:**
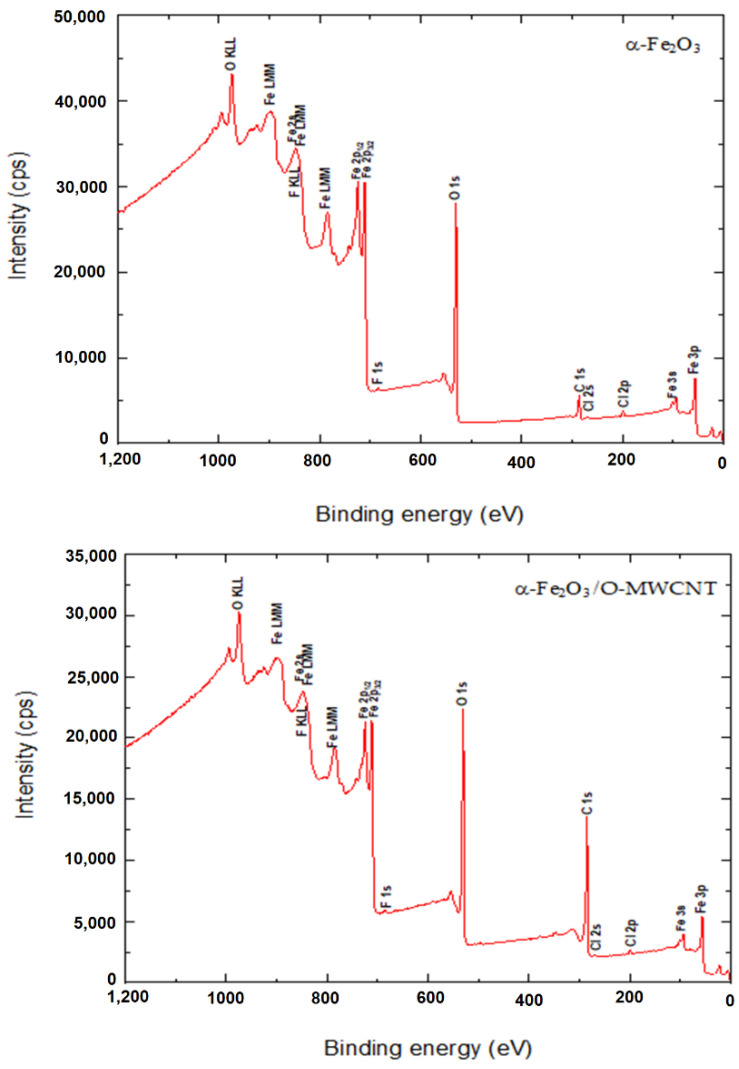
The wide-scan XPS survey spectra α-Fe_2_O_3_ and α-Fe_2_O_3_/O-MWCNTs composite.

The surface binding state and elemental speciation of α-Fe_2_O_3_ and α-Fe_2_O_3_/O-MWCNTs composite were analysed by XPS, and the wide-scan XPS survey spectra are presented in Figure 4. The XPS survey spectrum of α-Fe_2_O_3_ showed sub-peaks at binding energies 711.3, 714.6, and 718.8 eV, as well as their corresponding Fe2p1_1/2_ sub-peaks, corresponding to Fe^3+^ in α-Fe_2_O_3_ NPs; these sub-peaks are consistent with the oxygen bonds indicated as O1s A, O1s B, and O1s C at binding energies 529.6, 530.7, and 532.3 eV, respectively [33]. In addition, the presence of an insignificant amount of carbon and chlorine within the α-Fe_2_O_3_ sample may be attributed to the preparation process [33]. The XPS survey spectrum of the α-Fe_2_O_3_/O-MWCNTs composite and the binding energies for the C 1s and O 1s peaks were observed at approximately 284.5 and 531.0 eV, respectively, corresponding to the carbon and oxygen of the O-MWCNTs [34]. In addition to the sub-peaks of the α-Fe_2_O_3_, there were no signs of any other impurities in both samples according to the XPS analysis.

### 3.2. Electrochemical Measurements

**Figure 5:**
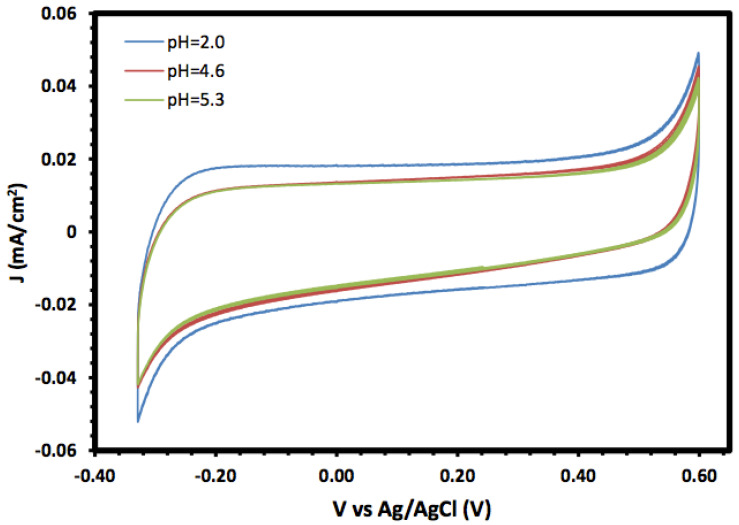
Cyclic voltammetry of α-Fe_2_O_3_ nanoparticles at different pH values.

The prepared α-Fe_2_O_3_ nanoparticles and its nanocomposite were investigated as an electrode material for supercapacitors, and the electrochemical properties were investigated using cyclic voltammetry (CV) and linear sweep voltammetry (LSV). Figure 5 shows the cyclic voltammograms of the prepared α-Fe_2_O_3_ in a 1.0 mol/L KOH solution at different pH values in the potential window range of −0.3 to 0.6 V. In general, the CV of the prepared α-Fe_2_O_3_ nanoparticles exhibited a rectangular shape without redox peaks, indicating the pseudocapacitive behaviour with fast and reversible surface reactions and good capacitive characteristics, which suggests that the α-Fe_2_O_3_ nanoparticle electrode is an excellent candidate for electrochemical double-layer capacitors. The consistent CV also indicated that the prepared α-Fe_2_O_3_ nanoparticles exhibited regular capacitive behaviour and excellent cycling stability [35,36].

Moreover, the voltammograms showed an increase in the current density, especially for the cathodic current, with lower pH values which may be due to the reductive dissolution of the α-Fe_2_O_3_ surface [37]. Moreover, the changes in the anodic current with pH were not significant compared with the cathodic current, and they may result from the oxidation of Fe(III) to Fe(IV) [38].

**Figure 6:**
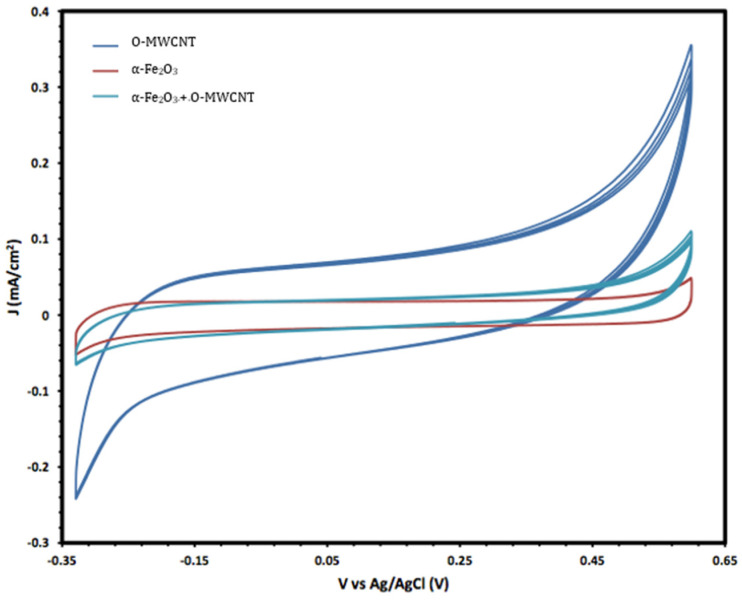
Cyclic voltammetry of O-MWCNT, α-Fe_2_O_3_ nanoparticles, and α-Fe_2_O_3_ /O-MWCNTs nanocomposite (1.0 mL O-MWCNT) in 1.0 M KOH.

**Figure 7:**
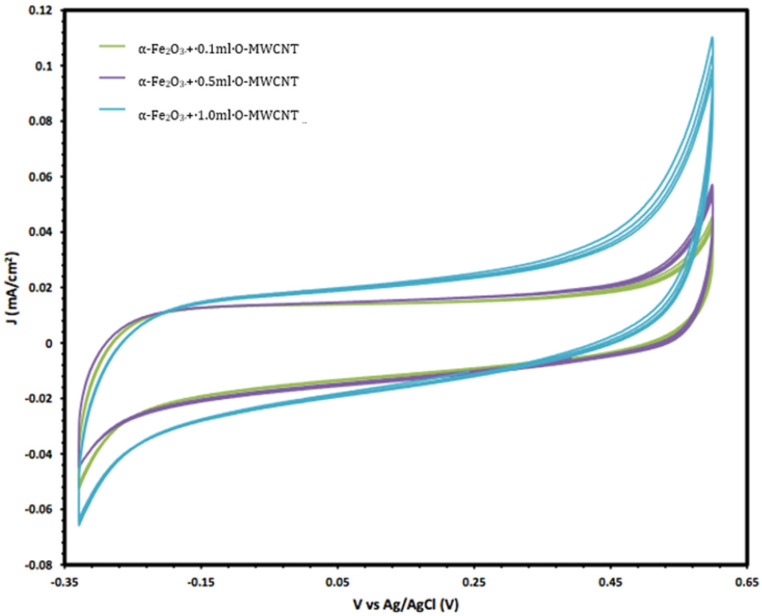
The variation in the cyclic voltammetry of α-Fe_2_O_3_ /O-MWCNTs nanocomposites with different amounts of O-MWCNTs.

Moreover, the electrochemical behaviour of the α-Fe_2_O_3_ nanoparticles in the absence and presence of O-MWCNTs was studied, and the results are presented in Figure 6. The voltammogram of the O-MWCNTs showed typical double-layer behaviour, and the featureless CV probably resulted from the distribution of the nanotubes, as well as the variations in length, diameter, and helicity of the arrangement of carbon hexagon rings at the working electrode [39]. Conversely, the voltammogram of the α-Fe_2_O_3_/O-MWCNTs nanocomposite showed the same characteristics as the pseudocapacitive behaviour, which were similar to α-Fe_2_O_3_, but with higher current density, especially for the cathodic current, which may indicate the enhancement of the reductive dissolution of the α-Fe_2_O_3_ surface in the presence of the O-MWCNT. The total capacitance is a result of α-Fe_2_O_3_ pseudocapacitance and EDLC capacitance of the O-MWCNTs [40,41]. Moreover, the variation in the O-MWCNTs within the α-Fe_2_O_3_/O-MWCNTs nanocomposite had a significant effect, as is presented in Figure 7, as the current density increased when increasing the amount of O-MWCNTs within the α-Fe_2_O_3_/O-MWCNTs nanocomposite, which may attribute to the enhancement of the specific surface area of the α-Fe_2_O_3_ electrode upon mixing with more carbon nanotubes.

**Figure 8:**
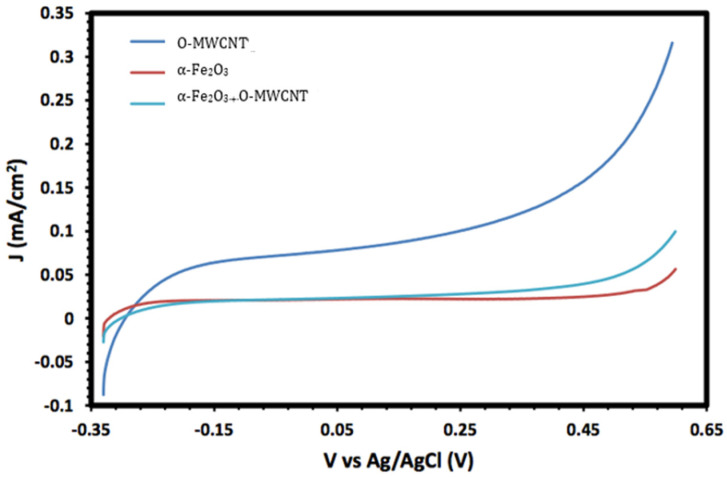
Linear sweep voltammograms (LSV) of O-MWCNT, α-Fe_2_O_3_ nanoparticles, and α-Fe_2_O_3_/O-MWCNTs nanocomposite (1.0 mL O-MWCNT) in 1.0 M KOH.

Figure 8 shows the linear sweep voltammograms (LSV) of O-MWCNT, α-Fe_2_O_3_ nanoparticles, and α-Fe_2_O_3_/O-MWCNTs nanocomposite (1.0 mL O-MWCNT) in 1.0 M KOH. It is clear from the figure that the generated current of the α-Fe_2_O_3_ nanoparticles are greatly enhanced upon the addition of the O-MWCNT, from 5.5 × 10^−2^ to 9.6 × 10^−2^ mA/cm^2^ for the α-Fe_2_O_3_ nanoparticles and α-Fe_2_O_3_/O-MWCNTs nanocomposite, respectively, which is almost 1.74 times higher, indicating the improvement of photoelectrochemical (PEC) performance. This enhancement in PEC performance could be due to the change in the morphology and hydrophilicity of the interface and consequently the faradic current upon the addition of the O-MWCNT, which is presented in Figure 8, as the charging current enhancement greatly depends on the amount of O-MWCNTs loaded.

**Figure 9:**
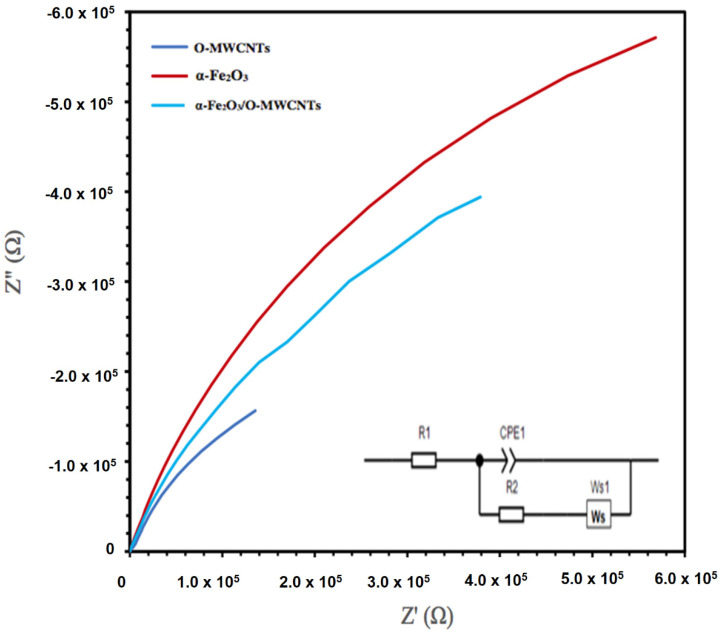
EIS Nyquist plots of O-MWCNT, α-Fe_2_O_3_ nanoparticles, and α-Fe_2_O_3_/O-MWCNTs nanocomposite (1.0 mL O-MWCNT) in 1.0 M KOH (inset is the proposed equivalent circuit).

The electrochemical impedance spectroscopy (EIS) of the hematite and its composite with O-MWCNTs was explored, as the EIS measures the opposition of alternating current flow of various frequencies applied to an electrochemical cell, such as a hematite body in contact with an aqueous solution. Figure 9 presents the Nyquist plot of the α -Fe_2_O_3_, O-MWCNTs, and α -Fe_2_O_3_/O-MWCNT. The inset in Figure 8 is the suggested equivalent circuit. The fitting results for α -Fe_2_O_3_, O-MWCNT, and α -Fe_2_O_3_/O-MWCNTs are summarized in Table 1. Rs is the solution/electrolyte resistance, which includes the contact and charge transfer resistances at the counter electrode/electrolyte (electrode interface) [42]. Rct is the charge-transfer resistance, CPE is the constant phase element, which represents the double-layer capacitance, and Ws is the Warburg impedance, which corresponds to the diffusion of the reactive species at the surface of the electrodes. The contribution of the electrochemical behaviour of the α -Fe_2_O_3_/O-MWCNTs nanocomposite is identified by the decrease in Rct and the increase in the double-layer capacitance in the presence of the O-MWCNT. The value of the charge-transfer resistance Rct for the a-Fe_2_O_3_ electrode decreases from 9454.9 Ω to 7236 Ω upon the addition of the O-MWCNTs and the formation of the α -Fe_2_O_3_/O-MWCNTs composite, while the value of CPE increases from 4.49 mF × cm^−2^ before adding O-MWCNTs to 5.53 mF × cm^−2^ after adding O-MWCNTs. The faster charge transfer is in the α -Fe_2_O_3_/O-MWCNTs nanocomposite, which may be attributed to an enhancement in the electrochemical behaviour and conductivity of the α -Fe_2_O_3_/O-MWCNTs nanocomposite.

**Figure 10:**
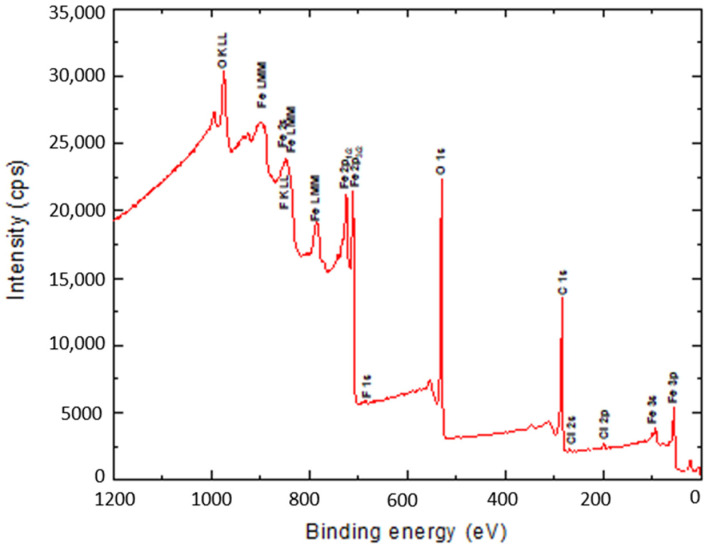
The XPS survey spectrum of the α-Fe_2_O_3_ and α-Fe_2_O_3_/O-MWCNTs composite after the electrochemical measurements.

Finally, the chemical stability of the α-Fe_2_O_3_/O-MWCNTs composite after the electrochemical measurement was explored using the XPS measurement, and the results revealed no change in the XPS survey after the electrochemical measurement (Figure 10), indicating the chemical stability of the α-Fe_2_O_3_/O-MWCNTs composite.

## 4. Conclusions

The α-Fe_2_O_3_/O-MWCNTs nanocomposite was successfully prepared using wet chemistry, and it was then characterized chemically and physically using different characterization techniques. UV–Vis absorption spectroscopy showed that the absorption of the colloidal α-Fe_2_O_3_ begins below 560 nm, indicating that the prepared α-Fe_2_O_3_ is a visible-light-active photocatalyst, and the FT-IR measurements showed the successful oxidation of the MWCNTs and preparation of the α-Fe_2_O_3_, as well as the α-Fe_2_O_3_/O-MWCNTs nanocomposite through the presence of their characteristic vibration peaks, whereas the XRD measurements showed the characteristic diffraction peaks of graphitic carbon nanotubes. The electrochemical behaviour of the α-Fe_2_O_3_/O-MWCNTs nanocomposite was investigated using cyclic voltammetry (CV), linear sweep voltammetry (LSV), and electrochemical impedance spectroscopy, and the results revealed that modification of the α-Fe_2_O_3_ nanoparticles with O-MWCNTs greatly enhanced electrochemical performance, conductivity and capacitive behaviour, as well as cycling stability.

## Figures and Tables

**Table 1:** Electrochemical double-layer capacitance, charge transfer resistance and solution resistance of α-Fe_2_O_3_, MWCNT, and α-Fe_2_O_3_/MWCNT.

	C_dl_ (mF cm^−2^)	R_ct_ (Ω)	R_s_ (Ω)
**Fe_2_O_3_**	4.49	9454.9	0.51
**Fe_2_O_3_/0.5 mL O-MWCNT**	5.53	7236	0.49
**MWCNT**	9.49E	4656	0.64

## Data Availability

Not applicable.

## Sample Availability

Samples of the compounds are available from the authors.
